# Cholesterol Organization in Phosphatidylcholine Liposomes: A Surface Plasmon Resonance Study

**DOI:** 10.3390/ma5112306

**Published:** 2012-11-13

**Authors:** Kathryn A. Melzak, Shirley A. Melzak, Electra Gizeli, José L. Toca-Herrera

**Affiliations:** 1 Department of Nanobiotechnology, Institute for Biophysics, University of Natural Resources and Life Sciences Vienna (BOKU), Muthgasse 11/ll, Vienna A-1190, Austria; E-Mails: kathryn.melzak@boku.ac.at (K.A.M.); s.melzak@boku.ac.at (S.A.M); 2 Department of Biology, University of Crete and Institute of Molecular Biology and Biotechnology, F.O.R.T.H., 100 N. Plastira Street, Crete 70013, Greece; E-Mail: gizeli@imbb.forth.gr

**Keywords:** cholesterol, cyclodextrin, liposome, membrane, membrane lateral organisation, OPPC, POPC, solubility, superlattice, surface plasmon resonance

## Abstract

Models for the organization of sterols into regular arrays within phospholipid bilayers have been proposed previously. The existence of such arrays in real systems has been supported by the fact that concentration-dependent sterol properties show discontinuities at the cholesterol mole fractions corresponding to regular lattice arrangements. Experimental results presented here are based on a surface plasmon resonance assay that was used to analyze rates of cyclodextrin-mediated removal of cholesterol from adsorbed liposomes at cholesterol mole fractions up to *χ_C_* = 0.55. Two kinetic pools of cholesterol were detected; there was a fast pool present at *χ_C_* > 0.25, and a slow pool, with a removal rate that was dependent on the initial χ_C_ but that did not vary as *χ_C_* decreased during the course of one experiment. The cholesterol activity therefore seems to be affected by sample history as well as local concentration, which could be explained in terms of the formation of superlattices that are stable for relatively long times. We also describe a variation on the traditional lattice models, with phosphatidylcholine (PC) being treated as an arrangement of hexagonal tiles; the cholesterol is then introduced at any vertex point, without increasing the total area occupied by all the lipid molecules. This model is consistent with Langmuir trough measurements of total lipid area and provides a simple explanation for the maximum solubility of cholesterol in the PC bilayer.

## 1. Introduction

Biological membranes typically consist of bilayers of amphipathic molecules, oriented so that their hydrophilic head groups are towards the aqueous surroundings, and their longer hydrophobic tails are in the membrane interior. Phospholipids, which are the most abundant of these membrane molecules, have a cylindrical shape, with the cross-sectional areas of the hydrophilic and hydrophobic portions being roughly equal. This permits the phospholipids to pack into the flat sheets of the bilayers. Cholesterol, another important component of cell membranes, does not form bilayers by itself, but will dissolve readily in the phospholipid bilayer. 

Models for the lateral arrangement of lipids show a schematic view of the top surface of the lipid layer, with the lipids being represented by circles. Cholesterol has approximately half the cross-sectional area of phosphatidylcholines [1]; this size difference has been taken into account in traditional lattice models by considering the cholesterol as being in a lattice of acyl chains [2,3,4]. There are two acyl chains per phospholipid, so that the acyl chains and the cholesterol are indicated in these models by circles of equal size. In this traditional superlattice model, the acyl chains are represented by a hexagonal close-packed array of circles, as shown in Figure S1a of the supplementary information; the acyl chain circles are then replaced by circles of a different color to indicate the presence of cholesterol, as shown in Figure S1b.

**Figure 1:**
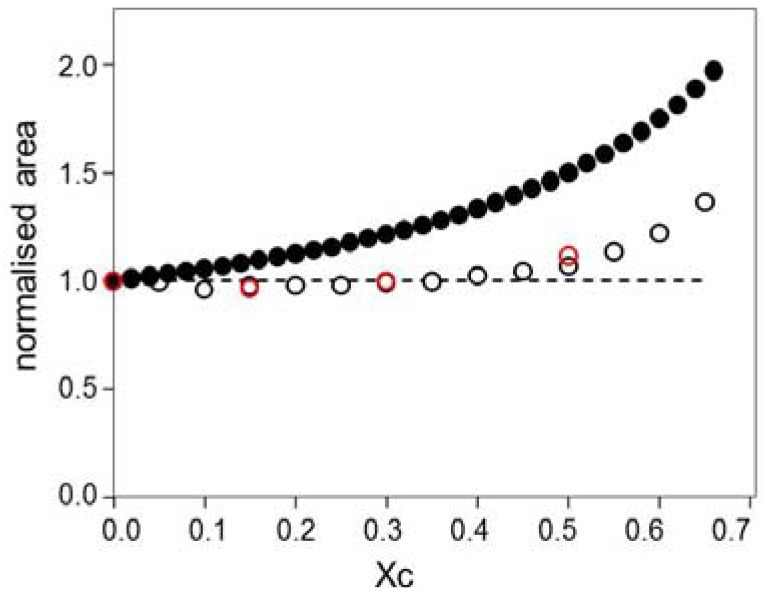
The relative increase in total lipid area is shown for a phosphatidylcholine (PC)-cholesterol mixture. Filled circles indicate the values that have been calculated assuming the model illustrated in Figure S1, and open circles indicate the approximate values that have been calculated based on Langmuir trough data published elsewhere (black circles [1]; red circles [5]. Calculations have been based on (a) the average area per molecule from Langmuir trough results, and (b) the relative increase in molecules present if cholesterol is added to a fixed amount of PC in order to obtain the desired *χ_C_*).

**Figure 2:**
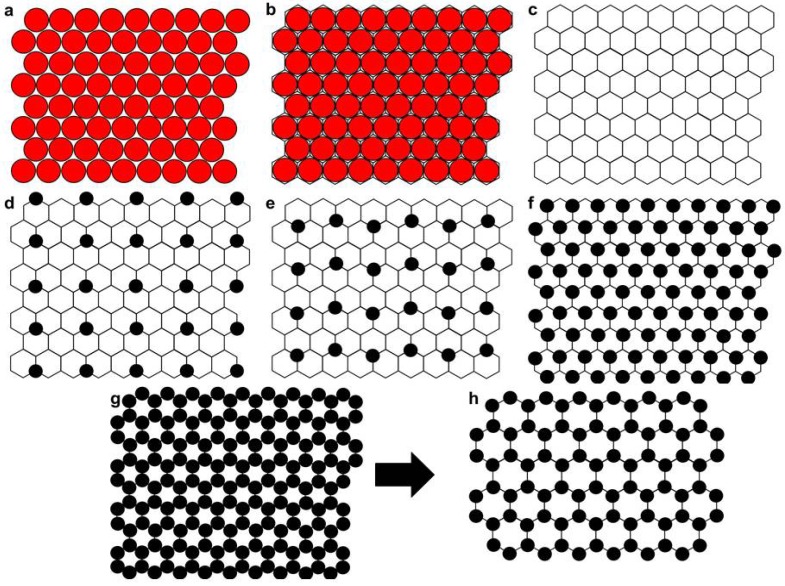
A model in which the cholesterol inserts without causing a change in the spacing between the PC molecules. The total area occupied by all the molecules does not change with increasing *χ_C_*. In (**a**), each circle represents one PC molecule; a hexagonal lattice may be drawn around the PC molecules, (**b**) the hexagonal lattice, (**c**) is then used for placement of the cholesterol molecules, shown in (**d**), (**e**) and (**f**) as black circles. Regular arrays are shown for *χ_C_* = 0.2 (d), 0.25 (e) and 0.5 (f). The model is expected to be reasonable up to *χ_C_* = 0.5, as described in the text. A regular array is also shown (**g**) at the solubility limit of *χ_C_* = 0.67 [6]; at this high a cholesterol content, the spacing between the PC molecules would be expected to increase, as indicated in Figure 1 and in (**h**). The relative sizes of the PC and the cholesterol do not affect the placement of the cholesterol in this model; cholesterol is drawn smaller here but without precise reference to scale. Both the PC and the cholesterol are represented here by circles. This will be a reasonable description for the PC, which is approximately cylindrical, but will not be an accurate description of the cholesterol. The cholesterol may be considered as an irregular shape that is placed between the PC molecules at positions indicated by the black filled circles.

For the model shown in Figure S1, it may be seen that addition of cholesterol to a fixed amount of phospholipid increases the total area occupied by the lipid molecules. The relative increase in area may be easily calculated. The first step in these calculations is to determine the amount of cholesterol that must be added in order to obtain the range of cholesterol mole fractions considered here, from *χ_C_* = 0 up to *χ_C_* = 0.67, the solubility limit of cholesterol in phosphatidylcholine [6]. As a sample calculation, if we start with 100 molecules of phospholipid, we must add 100 molecules of cholesterol in order to obtain *χ_C_* = 0.5, giving a total of 200 molecules present. The second step in the calculation was to determine the relative increase in area, based on two different assumptions about the molecular areas in the lipid layer. As mentioned above, traditional superlattice models assume that, at low cholesterol mole fractions (e.g., <33.3 mol%, [7]), a cholesterol molecule occupies half the area of a PC molecule. If we use this fixed ratio for our calculations, we obtain the results indicated in Figure 1 by the filled circles. The open circles in Figure 1 show values that have been calculated using experimentally determined molecular areas [1,5]. It may be seen in Figure 1 that the relative area increase differs substantially depending on the values that are used for the molecular areas. If we assume that the cholesterol occupies half the area of the PC, as in the traditional superlattice models, then the area increases with increasing χ_C_; if we use the experimental values for the area per molecule, however, we see that no increase in area is expected until the cholesterol reaches the relatively high mole fraction of 0.5. The Langmuir trough results therefore imply that the cholesterol inserts in the PC layer without causing an increase in spacing between the PC molecules. These results are in disagreement with the traditional superlattice models, as shown in Figure 1. We therefore suggest a modification to this model, as described below. Figure 2 illustrates a model in which cholesterol inserts into a PC layer without causing a change in the spacing between the PC molecules. This model is expected to be valid at cholesterol mole fractions lower than 0.5, but will be less accurate at very high cholesterol mole fractions. One way to accommodate the increased spacing that is expected between the PC molecules is to change the lattice dimensions. Previous superlattice models have also suggested changing the size of the circles used to represent the PC acyl chains [7].

One disadvantage of the Langmuir trough results is that they are based on measurements of monolayers at an air-water interface, rather than on measurements of lipid bilayers. It is certainly possible for lipids to behave differently in monolayers, one example of this being the fact that cholesterol has limited solubility in bilayers [6], but can be present up to *χ_C_* = 1 in monolayers [1,5]. The molecular areas determined from the Langmuir trough measurements may therefore differ slightly from the areas occupied within a bilayer. The Langmuir trough does, however, remain the standard instrument for determination of the cross-sectional areas of lipids.

The model shown in Figure 2 provides lattices equivalent to those of the standard superlattice model, in which regular arrays of cholesterol are proposed for various cholesterol mole fractions based on the geometry of the lattice arrangements. Regular arrays are shown in Figure 2 for *χ_C_* = 0.2, 0.25, 0.5 and 0.67; arrays may also be drawn for other cholesterol mole fractions (see supplementary information), including *χ_C_* = 0.4 and 0.57. The traditional lattice [4,7] that has been used to form the regular cholesterol arrays, as illustrated in Figure S1, can be used to produce patterns at all the cholesterol mole fractions mentioned here, but the patterns at the two highest values (*χ_C_* = 0.57 and 0.667) are not formed based on considerations of the geometric symmetry [2], a point which has been described as a failure of the traditional superlattice model [2] (the pattern at *χ_C_* = 0.57 is formed in this model by treating the cholesterol as dimers, while the pattern at the saturation limit is a maze rather than a repeating array). The modified lattice patterns shown here in Figure 2 and Figure S3 are therefore presented as a potentially useful addition to the superlattice theory.

The solubility limit of the cholesterol is explained here by a simple geometric argument: the limit of *χ_C_* = 0.67 in PC [6] is achieved by filling all the sites on the hexagonal lattice, as shown in Figure 2g and 2h. As noted in Figure 2h, this would require an increased spacing between the PC molecules, and thus between the lattice sites, in order to accommodate the cholesterol. This is in agreement with the Langmuir trough data described in Figure 1, which indicated that the relative area will increase above *χ_C_* = 0.5. 

One advantage of the superlattice model variation proposed here is that it matches more closely with the Langmuir trough data while still satisfying the requirements of the original model.

It is possible to draw models showing regular arrays of cholesterol at different mole fractions, but it is, of course, a different question to determine if such arrays exist in real samples. The early evidence for the formation of regular sterol arrays, or superlattices, came from the analysis of fluorescent sterols in bilayers [8]; since then, work on fluorescent sterols in phospholipid bilayers has also shown that there are dips in the concentration-dependent fluorescence intensity corresponding to the sterol mole fractions associated with regular sterol arrays [7]. The activity of enzymes acting on cholesterol-containing bilayers has also been shown to have discontinuities at the sterol mole fractions corresponding to regular arrays: this includes the activity of cholesterol oxidase, acting on cholesterol [2,9,10] and the activity of phospholipase, acting on the phospholipids in cholesterol-containing bilayers [11]. Evidence for sterol superlattice formation has been found in phospholipid bilayers containing both saturated [7,11,12] and unsaturated [2,9,11,12] acyl chains.

The effect of cholesterol mole fraction on the bilayer properties was investigated here by looking at the cyclodextrin-induced rate of removal of cholesterol from PC/cholesterol liposomes. This was done as a function of the initial mole fraction of cholesterol, in order to determine the effect of the initial *χ_C_*. Cyclodextrins, which are cyclic oligomers of glucose, have a hydrophobic core that enables them to take up hydrophobic molecules; they also have a hydrophilic exterior that keeps the whole complex soluble in water. The size of the hydrophobic cavity determines which molecules will be taken up. The β-cyclodextrins (βCDs) have seven glucose monomers and are able to form complexes with cholesterol (Chl); the βCDs have a high enough affinity for cholesterol that they are able to extract cholesterol from lipid bilayers. Complexes with a sufficiently high ratio of Chl: βCD will also be able to release cholesterol. The βCDs can thus be used to raise or lower membrane cholesterol content by using Chl: βCD complexes at suitable mole ratios, concentrations and application times [13,14]. In support of the model shown in Figure 2 we may note that addition of βCD to red blood cells, which have a membrane lipid composition of about 50 mol% cholesterol, causes a small increase in area rather than a decrease [15].

The kinetics of the cholesterol removal from liposomes were analysed here with surface plasmon resonance (SPR) [16] measurements on layers of adsorbed liposomes, following an assay procedure that has been described previously for studying the kinetics of cholesterol extraction [17]. The adsorbed liposomes were saturated with cholesterol prior to the start of the kinetics studies using a complex of cholesterol and methyl-β-cyclodextrin (MβCD:Chl); this permitted us to investigate liposomes at cholesterol mole fractions up to 0.55, moderately high relative to the solubility limit of 0.67.

## 2. Results

### 2.1. Cholesterol Removal and the SPR Response 

The purpose of these experiments was to analyze the kinetics of cholesterol removal from liposomes with initial cholesterol mole fractions up to the saturation limit of cholesterol in phosphatidylcholine bilayers.

The experimental format was that solutions of βCD were added to layers of adsorbed liposomes with a range of *χ_C_* values; the drop in the SPR signal was then monitored as a means of following the rate of cholesterol removal. The first requirement was therefore to determine the relationship between the drop in the SPR response and the removal of cholesterol. 

**Figure 3:**
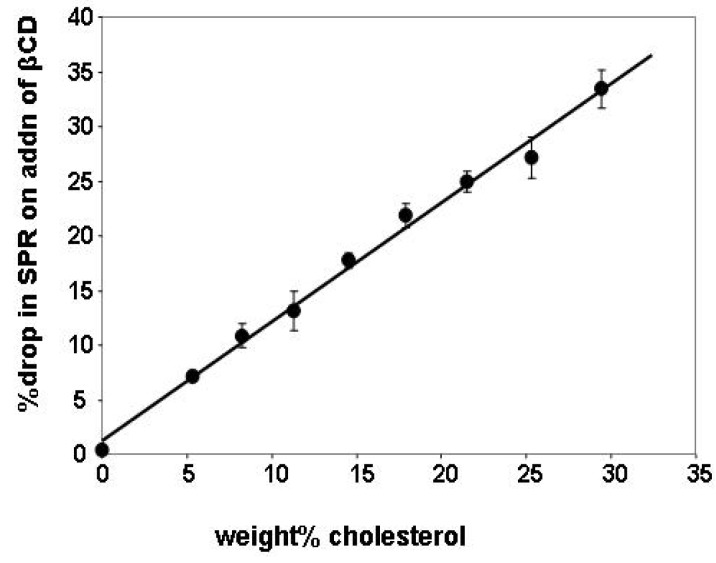
Relative drop in surface plasmon resonance (SPR) signal on βCD addition as a function of the cholesterol composition of the adsorbed liposomes; the straight line is described by the equation *y* = 1.086*x* + 1.305 (R^2^ = 0.993). Results represent averages from four different experiments.

The initial SPR response determined after deposition of liposomes was compared to the response after addition of βCD (determined after exchanging the βCD solution for buffer). The relative drop in signal was calculated as a percentage of the initial response for liposomes with a range of values of χ_C_ and plotted as function of the weight % cholesterol in the liposomes (Figure 3). The weight% was used here instead of the mole fraction as the independent variable because it is the weight% value that would be expected to be proportional to the refractive index, and hence to the SPR response.

Figure 3 indicates that βCD does not remove significant amounts of PC, that the results are reproducible in different experiments, and that the response is linear. Addition of the βCD to liposomes with no cholesterol caused a drop of only 0.4% in the signal, indicating that the βCD is specific for the cholesterol. Removal of the cholesterol may be associated with a proportional increase in the removal of the PC, or, alternatively, with desorption of the liposomes from the surface of the SPR slide; this would provide a possible explanation for the fact that the slope in Figure 2 is slightly greater than 1.

The results shown in Figure 3 were used to support the later analysis in which the drop in the SPR signal was assumed to be directly proportional to the decrease in the weight% cholesterol. The changing value of *χ_C_* during the course of the βCD addition was calculated by taking the initial response on addition of the liposomes, as before, and comparing it to the SPR response measured as a function of time during the βCD addition, after correction to account for the change in the bulk value of the refractive index associated with the βCD (this was determined from the SPR response on addition of βCD to liposomes with no cholesterol). Representative raw data for a cycle of liposome deposition and βCD addition on the modified surface of the SPR slide is shown in the supplementary data, in Figure S2a. Figure S2b also shows that signals associated with a series in which liposomes having *χ_C_* values from 0 to 0.35 were deposited on the SPR slides, and the cholesterol was removed with βCD.

### 2.2. Insertion of Chl with MβCD:Chl 

**Figure 4:**
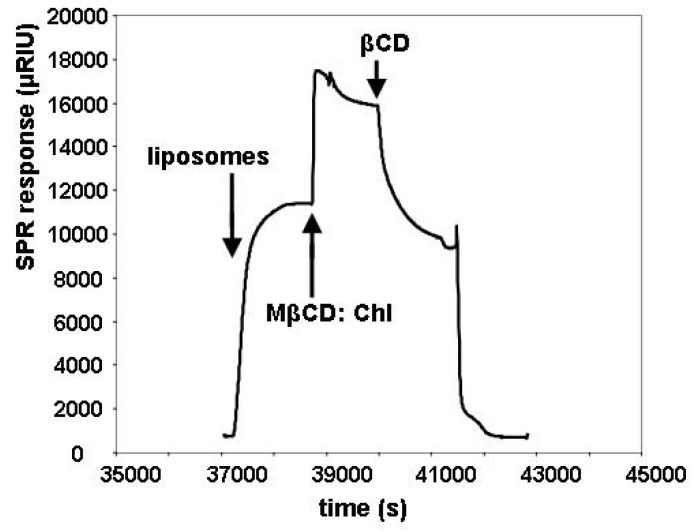
Representative raw data for a cycle of liposome deposition (*χ_C_* = 0.35), MβCD: Chl addition and βCD addition on the modified surface of the SPR slide. The addition times for these solutions are indicated by arrows on the Figure. The βCD removes both the cholesterol added via the MβCD: Chl and the cholesterol initially present, so that the final signal before the detergent rinse is lower than the initial signal.

The SPR response equilibrated rapidly on addition of the MβCD:Chl complex, as shown in Figure 4. Subsequent addition of βCD was used to determine the maximum cholesterol mole fraction that could be incorporated, based on the calibration curve shown in Figure 3. Liposomes with an initial *χ_C_* of 0 and 0.35 were used interchangeably for the insertion of additional cholesterol; the initial *χ_C_* had no effect on the proportional drop in signal observed on subsequent addition of βCD. The values calculated for the maximum χ_C_ were found to be 0.57 ± 0.03 for liposomes made with 1-palmitoyl-2-oleoyl-*sn*-glycero-3-phosphocholine (POPC) and 0.52 ± 0.03 for liposomes made with the POPC isomer OPPC, in which the positions of the two acyl chains are reversed. 

### 2.3. Kinetics of Cholesterol Removal from Liposomes with *χ_C_* ≤ 0.35 

**Figure 5:**
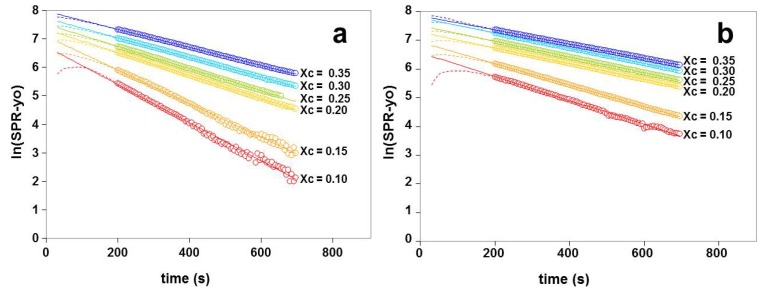
Demonstration of single exponential fits for removal of cholesterol from (**a**) POPC/cholesterol liposomes and (**b**) OPPC/cholesterol liposomes. For the POPC/cholesterol liposomes, the correlation coefficients for linear fits are 0.9999, 0.9996, 0.9996, 0.9997, 0.994, 0.993 for *χ_C_* values of 0.35, 0.3, 0.25, 0.2, 0.15 and 0.1, respectively; for the OPPC/cholesterol liposomes, the values are 0.9999, 0.9998, 0.9999, 0.9999, 0.9998 and 0.997 for the same sequence. Open circles indicate data used for linear fit; dashed lines indicate data outside region used for calculations; solid lines indicate linear fit.

The kinetics of the βCD-induced removal of cholesterol from adsorbed liposomes was analyzed and was found to follow a single exponential decay according to *y* = *y_o_* + Ae^−(*x*/*t*)^, where y is the measured SPR response, *y_o_* is the response with all the cholesterol removed, A is the additional response due to the cholesterol initially present in the liposomes, *x* is the time in seconds, and *t* is the time constant in seconds describing the rate of exponential decay. As can be seen from Figure 5, plotting ln(y − y_o_) against time (*x*) produces a straight line; the slope is equal to −1/*t* and the intercept is equal to lnA. It may also be noted, however, that data following a double exponential decay with two decay constants *t*_1 _and *t*_2_ would also give the appearance of single exponential decay at greater times, provided that *t*_2_ >> *t*_1_. If we assume that the removal of cholesterol follows exponential decay from time *x* = 0, then the initial rate of removal may be calculated from −A/*t*.

The mole fraction of cholesterol present decreases during the course of one measurement, because the cholesterol is removed by the βCD. The rate of cholesterol removal remained constant during this process, as indicated by the linear responses shown in Figure 5. In contrast to this, however, Figure 5 shows that the rate of cholesterol removal was affected by the initial χ_C_; the slopes are greater for liposomes with lower initial values of χ_C_, indicating that the cholesterol is more easily removed from liposomes with lower initial amounts of cholesterol present. The experiment series in Figure 5a, with POPC, was repeated with liposomes prepared from OPPC, as shown in Figure 5b. These two lipids are positional isomers and therefore have the same molecular weight and would be expected to have similar cross sectional areas. The interactions with cholesterol would be expected to be similar but not identical, as mentioned below in Section 3.2. The relative drop in signal on addition of βCD (as indicated in Figure 3) is the same for liposomes prepared from POPC and OPPC. The changes in the SPR signal may therefore be compared directly.

**Figure 6:**
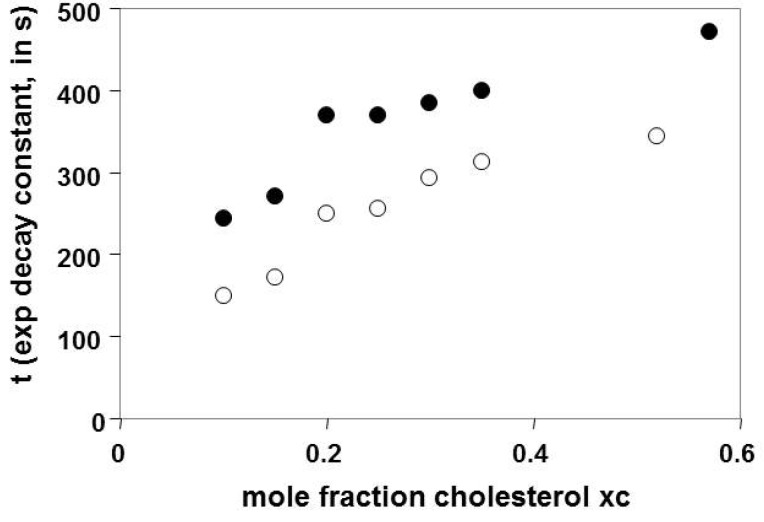
The exponential decay time constants for cholesterol removal from the liposomes are shown as a function of the initial cholesterol mole fraction in liposomes made from OPPC (●) and POPC (○). The time constants (*t*) refer to the single exponential decay observed on addition of βCD to adsorbed liposomes; larger values for *t* indicate slower removal of the cholesterol. The time constants are also shown here for liposomes saturated with cholesterol using MβCD: Chl; values are for the slow pool of cholesterol, as described below. The half-life for cholesterol in the sample is given by *t*ln2, and therefore ranges here from 100 to 330 s.

The cholesterol was removed from the POPC liposomes at a slightly greater rate than from the OPPC liposomes, but the change in the time constant describing the rate of cholesterol removal followed the same patterns with respect to the initial χc values, as shown in Figure 6. In both cases, there was a discontinuity around the initial cholesterol mole fraction of 0.2. The initial rate of cholesterol removal, which may be estimated as described in Section 2.3, increases with increasing *χ_C_* (not shown).

**Figure 7:**
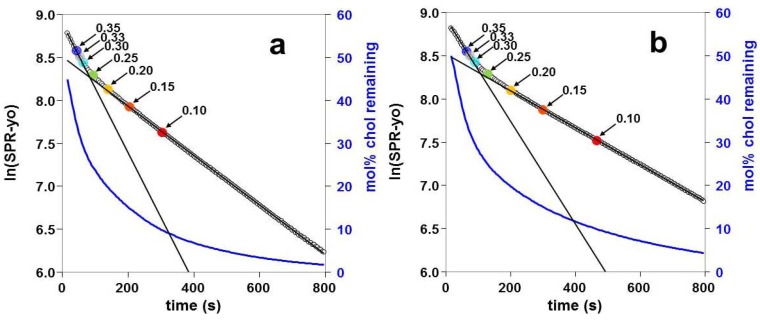
Kinetics of cholesterol removal from (**a**) POPC and (**b**) OPPC liposomes saturated with cholesterol. The half-life times for slow pool cholesterol are 240 s (POPC) and 325 s (OPPC). As for Figure 5, *y_o_* is the SPR response with all the cholesterol removed; other terms in the exponential decay equation are as defined above in Section 2.3. The calculated mol% of cholesterol remaining in the liposomes is shown by the line in dark blue (see right-hand axis). The calculated *χc* remaining in the adsorbed liposomes is indicated on the Figure for the filled colored circles; colors correspond to those in Figure 5. Values used for these Figures represent averages of ten or more experiments; examples for individual experiments are shown in the supplementary information.

### 2.4. Kinetics of Cholesterol Removal from Liposomes Saturated with Cholesterol 

Liposomes that were saturated with cholesterol using MβCD: Chl were shown to have two kinetic pools of cholesterol, as illustrated in Figure 7. One pool was removed within the first 200 s, and a second pool was removed more slowly. The time constants calculated for the second pool using a single exponential fit followed the pattern observed for the change with respect to the initial *χ_C _*values, as shown above in Figure 6. The analysis of the kinetics was carried out as described in Section 4.7.

## 3. Discussion 

The points of interest in these results are that the sample history affects the rate of cholesterol removal; that the rate of removal varies with initial χ_C_; that it is possible to have two kinetic pools of cholesterol with only cholesterol and phosphatidylcholine present, and that there is a discontinuity in the rate of removal around *χ_C_* = 0.2–0.25. These results will be discussed separately and then in relation to the modified lattice model that was proposed in the introduction.

### 3.1. Effect of Sample History: Rate of Cholesterol Removal a Function OF Initial *χ_C_*

First, we will consider the fact that the rate of cholesterol removal is a function of the initial *χ_C_*, instead of being a function of the χ_C_ value at any given time during the course of one experiment. The results in Figure 5 and Figure 7 show that a single exponential decay model fits very well for data obtained at times greater than 200 s, indicating that the rate of cholesterol removal remains constant during this period. During the course of the βCD addition, however, all the cholesterol will be removed (see Figure S3b). If the initial *χ_C_* is high, then addition of βCD will result in the cholesterol content of the adsorbed liposomes passing through the entire range of values used here for initial *χ_C_*. In Figure 7, for example, it may be seen that when the *χ_C_* of the adsorbed liposomes drops below 0.1, the slope of the graph does not change to that observed for liposomes with an initial *χ_C_* value of 0.1. It is therefore apparent that the rate of cholesterol removal is affected by the initial concentration, and thus the sample history, and not just by the concentration of cholesterol at any given moment. 

These results imply that there is a time-dependent rearrangement of the liposomes that affects the ease of removal of cholesterol. This would be consistent with the slow formation of a lattice that is not disrupted over the relatively short time course of the experiment. It has been suggested that long equilibration times are required to permit the formation of lattices in lipid layers; procedures for preparation of vesicles for superlattice experiments have employed incubation times of hours [18] or days [2,7]. Variations in lattice formation due to sample incubation times or other factors are therefore a potential factor that may contribute to the diverse results [13] that have been obtained for βCD extraction of cholesterol.

It is, however, possible that a lattice arrangement of some sort is formed more rapidly when the liposomes are saturated with cholesterol, perhaps due to limitations in the cholesterol and phospholipid arrangements associated with the solubility limit. This is suggested by the results observed for the slow pool of cholesterol, when it is removed from liposomes that have been saturated with cholesterol by insertion via MβCD:Chl. The removal rate for this slow pool followed the trend displayed in Figure 5 and Figure 6; the liposomes behaved as if they had equilibrated at a higher initial χ_C_, even if the insertion of cholesterol occurred very rapidly. The maximum *χ_C_* values that were obtained were, for the POPC *χ_C_*_max_ = 0.57 ± 0.03, in comparison to the solubility limit of *χ_C_* = 0.67; the insertion into the OPPC liposomes resulted in a value that was slightly lower, but within the error limits for the measurements (0.52 ± 0.03). 

### 3.2. Time Constants in the Slow Pool Increase with Increasing *χ_C_*

The second feature of interest from the results is the manner in which the rate of cholesterol removal varies with the initial value of *χ_C_*. It can be seen in Figure 6 that the time constants increase with increasing *χ_C_*, for both PCs investigated here. The half-life for the cholesterol in the liposomes increases by a factor of three, to a maximum value of about 330 s. This corresponds to a decrease in the rate of removal of the cholesterol, possibly due to decreased mobility of the bilayer lipids (addition of cholesterol has been found to decrease the mobility of all regions of the POPC molecule [19], although the rates of lateral and rotational diffusion remain high [20]). The mechanism of βCD-induced extraction of cholesterol is not well characterized but is described as being likely to involve an initial step in which bilayer fluctuations cause a partial protrusion of the cholesterol into the aqueous phase adjacent to the bilayer [13]. A decrease in the lipid mobility within the bilayer would thus be expected to affect the rate of cholesterol removal by βCD. It would also be possible for the bilayer fluctuations associated with the protrusion of cholesterol into the aqueous phase to be decreased by the formation of arrays at higher χ_C_ values; if the lipid ordering associated with these arrays can persist over the time course of these experiment, this could explain the time-dependent results noted above. The effects of increased lipid mobility may also contribute to the increased rate of cholesterol removal in POPC liposomes relative to OPPC, due to the greater mobility of POPC in the headgroup region [21]. 

### 3.3. Two Kinetic Pools of Cholesterol in PC/Cholesterol Mixtures

Figure 7 shows that there is an additional pool of cholesterol that is removed more rapidly at higher values of *χ_C_*. Experiments with βCD-induced removal of cholesterol have shown previously that cells have two pools of cholesterol that are removed at different rates [22]. Multiple kinetic pools have also been found for cholesterol in liposomes; one study found that sphingomyelin must be present in liposomes in order to produce two kinetic pools of cholesterol [17], while another study has found two kinetic pools in liposomes with no sphingomyelin [22]. The requirement for sphingomyelin that has been found elsewhere [17], which is in contrast to the results presented here, may have been due to the use of a different phospholipid, or else to the fact that the kinetic studies started at a cholesterol mole fraction of 0.3, unlike the work presented here. The rapidly removed pool of cholesterol may not be sufficiently large to be apparent unless the liposomes start with a relatively high mole fraction, as may be seen in Figure 7. Cholesterol arrangements and interactions that could account for the two pools are discussed below.

### 3.4. Discontinuity in the Time Constants Describing the Rate of Cholesterol Removal, Near *χ_C_* = 0.2–0.25

Figure 6 and Figure 7 both indicate a discontinuity in the rate of cholesterol removal. Although we are able to vary *χ_C_* systematically, we do not have either small incremental changes in the *χ_C_* values, or an independent determination of the *χ_C_*, as discussed in Section 4.4 describing the preparation of the liposomes; we are therefore only able to say that there is a discontinuity in Figure 6 and Figure 7 somewhere near *χ_C_* = 0.2 or 0.25. The effect is not very prominent on Figure 6, but was reproducible with different phospholipids, as seen by the comparison of results for POPC and OPPC. In our model, *χ_C_* = 0.25 corresponds to the lipid mole ratio at which every PC molecule is in contact with one molecule of cholesterol, as illustrated in Figure 2e, as is discussed in more detail below. Our results are consistent with results presented elsewhere, in which a prominent jump in the activity of cholesterol oxidase acting on cholesterol in POPC/cholesterol bilayers was noted at *χ_C_* = 0.25 [2].

It has also been demonstrated that the rate of sterol removal can show local maxima at sterol mole fractions corresponding to the ratios associated with the regular arrays [23]; a local maximum in the activity of cholesterol oxidase has also been observed at *χ_C_* = 0.25 [10]. The increased rate of sterol removal was attributed to facilitated removal of the sterols from the interfaces surrounding the areas with regular arrays; the increased lattice formation at these mole ratios produces a corresponding increase in the borders and is thus associated with an increased rate of sterol removal. Demonstration of these local maxima requires production of lipid mixtures with very small increments of the sterol mole fractions. This was not done for the work presented here but could be of interest for possible future experiments. It may also be noted that in the work cited here [23], the local maxima are superimposed on a background trend that shows a discontinuity near *χ_C_* = 0.2; the initial rates of sterol removal are relatively constant below this point, but increase at higher *χ_C_* values.

The discontinuity in Figure 6 and Figure 7 appears to reflect a change in the rate of cholesterol removal and not a change in the initial SPR response associated with the liposomes. No corresponding discontinuity was noted in the SPR response as a function of *χ_C_* of the deposited liposomes. Deposition of a series of liposomes with increasing values of *χ_C_* is illustrated in Figure S2b of the supplementary information; the net change in the signal is not summarized here, but has been reported elsewhere [24]. Analysis of deposited PC/cholesterol liposomes using an acoustic device that is sensitive to the mechanical properties of thin adsorbed layers has, however, shown a discontinuity at a cholesterol mole fraction slightly over 0.2 [24], implying that there is a structural change in the lipid layer at this ratio of cholesterol to PC.

### 3.5. Some Possible Structural Arrangements for PC/Cholesterol Bilayers 

Possible structural arrangements that could account for two pools of cholesterol include the two leaflets of the lipid bilayer, the co-existence of liquid ordered and liquid disordered phases, the formation of specific complexes, and the formation of arrays with cholesterol in positions that are not all equivalent. The flip-flop of cholesterol between the two leaflets of the lipid bilayer is rapid [25], implying that the inner and outer leaflets of the bilayer shell of liposomes are not responsible for the two pools of cholesterol. The separation of lipids into lateral domains has therefore been suggested as the cause for the two kinetic pools that have been observed in cells and liposomes [13,17,22]. Two kinetic pools have also been observed for enzymatic oxidation of cholesterol in cell membranes; it has been suggested that these two pools represent the co-existence of ordered arrays with the irregular regions [10].

Separation of the lipids into regions with different phases would be expected at 25 °C: for the POPC/cholesterol system, the lipids would be present in a homogeneous liquid disordered (ld) phase at *χ_C_* < 0.1, as both the ld and liquid ordered (lo) phases at 0.1 < *χ_C_* <0.4, and as homogeneous lo phase at 0.4 < *χ_C_* [26]. The co-existence of different phases would not explain the results seen here: the time-dependent results are not explained, and the discontinuities seen near *χ_C_* = 0.25 do not correspond to the start of lo formation at *χ* = 0.1 or to the formation of a homogenous lo phase at *χ_C_* = 0.4. The presence of increasing amounts of lo phase could explain the pattern observed in Figure 5, in which the rate of removal decreases with increasing initial *χ_C_*; this could be due to the slower removal of cholesterol from the less mobile lo phase. This would, however, be insufficient to explain the rapidly removed pool of cholesterol at higher *χ_C_* values, as seen in Figure 7. The formation of heterogeneous phases should be taken into consideration when analyzing other possible explanations for the observed results. Some points to consider are that mole fraction of cholesterol will be different in each of the two phases; the ld region has a lower cholesterol content than the lo region [27], and that the lattice formation could be limited to one phase, such as the lo phase. The value for χ_C_ in the ld phase would be slightly higher than that for the total lipid composition. Models for superlattice formation do not suggest that the lattices cover the entire membrane area [4,7], but do suggest that the lattice area is maximized at the relevant mole fraction [3,7].

One additional feature associated with increased cholesterol content is that isolated cholesterol-rich domains can become connected at sufficiently high *χ_C_* values. Monte Carlo simulations based on the mixing of cholesterol with sphingomyelins and dipalmitoyl phosphatidylcholine have suggested that the switch from isolated to connected domains can occur at *χ_C_* = 0.2; it has also been suggested that this change may be associated with an increase in the lateral diffusion constants [28]. It is certainly possible that increasing the cholesterol content in the lipid mixture used here could also result in connection of the isolated domains. As described in Section 3.2 above, the time constants increase with increasing initial *χ_C_*, indicating that cholesterol has a longer half-life within the bilayer. It is unclear why an increase in the lateral diffusion constant would cause a decrease in the cholesterol removal, and we will therefore consider alternate explanations of our results.

The cholesterol may also form specific complexes with phospholipids. For cholesterol interactions with bilayers, the sterol ring structure can insert into the hydrophobic portion of the bilayer, with the hydroxyl group of the cholesterol then forming a hydrogen bond with the doubly bonded oxygen of one of the phospholipid ester groups [29]. Studies of lipid interactions at air-water interfaces have led to the suggestion that cholesterol forms condensed complexes with some phospholipids at a 2:1 ratio of cholesterol to phospholipid, with up to 30 molecules in one complex [30]. The formation of complexes does not explain the results observed here, but is compatible with the possibility that the lipids can also form lattices [31]. Condensed complexes and lattices have been described by similar mathematical models, and the complexes have been described as being precursors to the formation of lattices [32].

### 3.6. Modified Lattice Model

The modified lattice model proposed in Figure 2 is similar to the original lattice model, with arrays that can be drawn at the same cholesterol mole fractions. In addition, the modified version of the model can be used to draw an array with hexagonal symmetry at *χ_C_* = 0.67, the solubility limit [6] for cholesterol in PC bilayers, as shown in Figure 2. This compares to the maze pattern that may be generated by Monte Carlo simulations for *χ_C_* = 0.67 [2,33] when using the original lattice model [7]. During the βCD-induced removal of cholesterol, the modification shown here in Figure 2 allows for a transition between the array formed at saturation and other regular arrays, without the requirement for rearrangements of the lipids. Regular arrays could thus be maintained during the cholesterol removal. In our results, this would explain why the rate of removal from the slow pool of cholesterol follows the trend observed in Figure 6. 

The formation of lattices is generally described as being slow [2,7,18,31]. At the solubility limit of cholesterol, however, the cholesterol and PC may be driven into a lattice arrangement, since the regular array would be required to accommodate the high mole fraction of cholesterol while avoiding the energetically costly [2] cholesterol clustering. This could provide a mechanism by which the insertion of cholesterol into liposomes, using the MβCD:Chl complex, could lead to a rapid formation of a regular array. 

The very good fit that was observed for single exponential decay at longer times implies that the extractable cholesterol is all equivalent. One possible explanation for these results would be that the arrays cover the entire surface of the liposomes, although this is described as being unlikely due to thermal fluctuations and impurities [7] (the arrays have been estimated to cover as much as 90% of the membrane surface area at critical sterol mole fractions [10,32,34], although the area covered can be much lower at *χ_C_* values slightly different from the critical ones [34]. 

An alternate possibility is that the cholesterol is present in different pools, but that extraction occurs exclusively from one pool during the time course followed in Figure 5. This would be the case if the cholesterol in a second pool were removed during the first 200s of the experiment, if the cholesterol were removed very slowly relative to the time course in Figure 5, or if the cholesterol could exchange between different pools over the course of the experiment, with the βCD-induced removal occurring from one pool only.

Experiments that have been used to obtain evidence for the presence of ordered arrays of sterols in lipid bilayers typically look for discontinuities in the lipid properties. These discontinuities are expected to be jumps [2] or else local minima and maxima [3,7,9,10,11,12,18,23,31] depending on the manner in which the arrays are being described. As mentioned above in Section 3.4, these discontinuities occur over narrow ranges of sterol mole fractions, so that the initial samples must be prepared with correspondingly small incremental increases in sterol concentration. In the experiments described here, the initial cholesterol mole fractions were prepared in widely spaced increments, so that we would not expect to see any clearly-defined maxima or jumps in the rates of cholesterol removal. The discontinuity observed in Figure 6 can therefore not be definitively associated with the local maxima such as those observed at *χ_C_* = 0.25 for the activity of cholesterol oxidase [10]. During the βCD removal of the cholesterol, the cholesterol content of the liposomes will be high at the start of the experiment, and close to 0 by the end; χ_C_ therefore passes through a range of values without any local maxima in the removal rates, or, indeed, without seeing any deviation from the single exponential decay, as described above. This is attributed here to the slow formation of the regular arrays [2,7,18,31]; although the liposomes will pass through all the cholesterol mole fractions associated with the regular arrays during the course of the experiments illustrated on Figure 5, there is insufficient time for new arrays to form.

## 4. Experimental Section 

### 4.1. Materials

1-oleoyl-2-palmitoyl-*sn*-glycero-3-phosphocholine (OPPC) purchased from Sigma and 1-palmitoyl-2-oleoyl-*sn*-glycero-3-phosphocholine was purchased from Fluka; both were used as received, and made up as stock solutions at 20 mg/ml in chloroform from Merck. Phosphate buffered saline (PBS) was prepared from tablets (Sigma) dissolved in water purified by distillation followed by deionization with a Barnstead NANOpure filtration system to obtain 0.01 M phosphate, 0.0027 M potassium chloride and 0.137 M sodium chloride pH 7.4. Medium molecular weight poly(methyl methacrylate) (PMMA) and 2-ethoxyethyl acetate (2-EEA) were purchased from Aldrich for modification of the SPR slide. Hydrochloric acid (HCl) from Merck and tetraethyl orthosilicate (TEOS) from Fluka were used to prepare a silicate layer modification for the SPR device; the SPR devices were regenerated with Triton X-100 detergent (Merck). The β-cyclodextrin was from Fluka; concentrations of βCD were calculated based on the dry weight, using the value for the water content provided by the manufacturer. Water-soluble cholesterol (MβCD:Chl) refers to cholesterol that has been prepared with an excess of methyl-β-cyclodextrin and was purchased from Sigma. (2-(12-(7-nitrobenz-2-oxa-1,3-diazol-4-yl)amino)dodecanoyl-1-hexadecanoyl-*sn*-glycero-3-phosphocholine (NBD C_12_-HPC) was purchased from Molecular Probes.

### 4.2. Modification of the SPR Slides with PMMA and Silicate 

Gold-coated slides were purchased from Xantec with a nominal gold thickness of 47.5 nm. The slides were then modified as described previously in order to induce adsorption of POPC liposomes. Slides were coated with a thin layer of PMMA by spin coating from a 1% solution of PMMA in 2-EEA. Solvent was removed from the adsorbed layer by heating the PMMA-coated slide to 195 °C for two hours. The slide was then modified with a layer of silicate gel prepared by condensation from TEOS in the presence of acid [35]. The TEOS (0.25 g) was added to a 1.5 mL microcentrifuge tube and 5.8 M HCl was added to give a final volume of 1 mL. The 2-phase mixture was left for 1 min before being vortexed at 1400 rpm for 2 min, so that the TEOS hydrolyzed and one phase was formed. The hydrolyzed TEOS mixture was left at room temperature until sufficient cross-linking had occurred that a bubble of air injected into the mix remained in place (this occurred at approximately 20 min after the start of the mixing process, and about 1.5 min after the mixture was sufficiently solid to remain in place when the tube was inverted). The TEOS mixture was then placed on the PMMA-coated SPR slide for 2 min before being rinsed off with water. The flow cell of the SPR instrument was put in place on the modified slide without letting the slide dry off. At the end of the experiments, the gold surface of the SPR slide was regenerated by rinsing the slide with acetone. 

### 4.3. Surface Plasmon Resonance 

Surface Plasmon Resonance (SPR) experiments were carried out with a Reichert SR 7000 instrument maintained at 25 °C, in a room with an ambient temperature of 25 ± 1 °C. The change in the position of the resonance minimum was measured as a function of time and was displayed in resonance units. Data was collected every 5 s. The SPR measurements are based on the refractive index change of the layer adsorbed on the SPR slide, which consists of a thin gold layer on a transparent material such as glass. The βCD-induced removal of cholesterol from the adsorbed liposomes will reduce the amount of lipid adsorbed to the surface, thus bringing the refractive index of the adsorbed layer closer to that of the surrounding medium and changing the SPR signal.

### 4.4. Liposome Preparation 

Unilamellar lipid vesicles were prepared by extrusion using an Avestin Liposofast Basic extrusion apparatus. Vesicles were made from either POPC or OPPC, mixed with cholesterol at various mole fractions cholesterol (*χ_C_*). Stock solutions of lipids were made up in chloroform and mixed to give the appropriate mole ratio. Chloroform was removed by evaporation under a stream of nitrogen for one hour. The dried lipids were resuspended in PBS at a total lipid concentration of 2 mg/mL and then extruded 25 times through a 50 nm pore membrane (Avestin). OPPC liposomes prepared by this procedure were shown previously to have a diameter of 70 nm [36]. Liposomes were then stored at 4 °C before use.

The values of *χ_C_* in the adsorbed liposome layer were calculated from the relative amounts of lipids that were mixed together during the liposome preparation. This method permits good precision in determining the values for *χ_C_*, but leads to potential questions about the accuracy. The observed results were found to be consistent with accurate preparation of liposomes having a range of *χ_C_* values, as shown in Figure 3. It has been reported previously [33] that de-mixing of the cholesterol can occur during the liposome preparation step, leading to inaccurate values for *χ_C_*. This problem has, however, been described as affecting only liposome preparations with high values of *χ_C_*, and did not appear to affect the results in these experiments.

The filter used during the extrusion process was found to trap 15% of the fluorescent PC, as shown by incorporation of 1% NBD C_12_-HPC, which was added to the lipid preparation when the stock solutions were mixed together. The fluorescence intensity was then determined for the initial aqueous suspension and for the extruded liposome preparation with a Synergy HT 96-well plate fluorimeter with *λ_ex_* = 385 nm. 

### 4.5. Formation of Vesicle Layers on Silicate 

A continuous flow of PBS was pumped over the surface of the modified SPR slides using a peristaltic pump. For the POPC/cholesterol liposomes, vesicle suspensions at a total lipid content of 0.4 mg/mL were pumped over the surface of the silicate-modified device for 15 min. The device was then rinsed with PBS for 15 min before addition of β-cyclodextrin (βCD) or insertion of cholesterol as described below. The surface was regenerated with a 5 min detergent rinse of 0.1% w/v Triton X-100 in PBS. Following a PBS rinse of 15 min, the device surface returned to its initial state so that an additional cycle of liposome deposition could be started. The interactions with the substrate do not appear to affect the rate of removal of the cholesterol: in work reported elsewhere, liposomes were also attached to the surface via a tether, and the βCD- induced removal of cholesterol was monitored using an acoustic device, and the rate of removal of the cholesterol was found to be unaffected by the mode of attachment [36].

### 4.6. Addition of β-Cyclodextrin and MβCD:Chl 

A solution of βCD was added to liposome layers on the silicate-coated SPR slides after the liposomes had been rinsed with buffer for 15 min. The βCD solution had a concentration of 5.2 mM (6 mg/mL) and was added for 30 min, followed by a 5 min buffer rinse and addition of detergent to regenerate the surface. Longer addition times of βCD did not lead to an increase in the % drop in the SPR signal associated with the βCD addition. A brief pulse of rapid flow of buffer was typically employed to clear the tubing. This would have the potential to dislodge some liposomes from the surface; such desorption should not, however, affect the results obtained for the relative change in signal on addition of βCD since the values of interest are the SPR response before and after the βCD addition.

Cholesterol was inserted into adsorbed liposomes by addition of a commercial preparation of cholesterol and MβCD. The MβCD:Chl was used at a relatively high concentration of 1.8 mg/mL in order to minimize the formation of precipitate, which occurred during dilution. Some precipitate was formed in the plastic tube leading to the flow cell over the SPR slide. This is consistent with other observations that the complexes are not stable when stored in plastic [14]. 

### 4.7. Analyzing Kinetics of Cholesterol Removal 

The kinetics of cholesterol removal were analyzed via a two part procedure. Initially, the decay in the SPR signal on addition of βCD was analyzed with the graphing program Origin to determine the value *y_o_* for the single exponential decay described by *y* = *y_o_* + Ae^−(*x*/*t*)^, where *y* is the SPR signal, *x* is the time in s, *t* is the decay constant in s, *y_o_* is the SPR at infinite time (that is, the signal when all the cholesterol is removed) and A is the extent to which the initial SPR signal increases due to the presence of cholesterol. The zero-time for the addition of βCD was taken to be the time at which the bulk solution in the SPR flow cell reached its maximum refractive index value; this time was calculated based on results obtained for the addition of βCD to adsorbed liposomes with no cholesterol (for this sample, the SPR response on addition of βCD will be due to the refractive index change of the bulk of the solution and will not have a significant component associated with the change in the adsorbed layer). For liposomes with *χ_C_* > 0, some cholesterol removal will take place before the βCD solution has reached its maximum concentration. The rate of cholesterol removal is a function of the βCD concentration [17], and will therefore vary over the period before the solution concentration of βCD equilibrates. These problems could be minimized by the use of a smaller flow cell, but smaller flow cells and narrow tubing would have created problems with the MβCD: Chl, which tends to form predicates, as mentioned above in Section 4.6. It would be possible for cholesterol to precipitate on the surface of the modified slide, thus providing an additional source of cholesterol to be removed by the βCD but the consistent results with the maximum *χ_C_* reached as expected, and two smooth single exp decay curves imply that there are no problems with precipitate formation.

The second part of the kinetics analysis was to use the values obtained for y_o_ and plot the data in a format that would linearize data if it followed a single exponential decay; this involved plotting ln(*y* − *y_o_*) against time. The linear format of the graphs permits a quick visual inspection of the quality of the fit; an additional benefit is the ease with which one can distinguish the existence of two separate decay constants. The linearized results from multiple experiments were then averaged to produce the results shown here; the decay constants were calculated from the slopes. Attempts were also made to obtain a bi-exponential fit to the data, also using the graphing program Origin; this provided good fits but with negative values for A. These solutions were therefore discarded. 

## 5. Conclusions 

Our model is compatible with other results that have been presented in support of cholesterol lattice formation within phospholipid bilayers, in the sense that regular arrays can be produced at defined mole fractions. One advantage of the modification that we are suggesting is that it corresponds more closely to the well-established Langmuir trough data regarding the mixing of phospholipids and cholesterol; an additional advantage is that it provides a simple way to explain the cholesterol solubility limit. 

A regular array can be produced at the solubility limit based only on considerations of the symmetry. Some of the elements in this array are at the same relative locations as those in arrays formed at lower cholesterol content, leading to the possibility of smooth transitions between arrays at high and lower cholesterol mole fractions.

The experimental results obtained here are consistent with the removal of cholesterol from a lattice that has a slow equilibration time, as has been suggested elsewhere [2,7,18] and that is not able to rearrange itself during the time course of one experiment. Our results show that cholesterol removal at lower mole fractions corresponds closely to a single exponential decay, implying that all the cholesterol is equivalent; this could be due to formation of arrays that cover a high proportion of the surface, as can happen at critical sterol mole fractions [10,32,34]; alternatively, the cholesterol could exist in different pools, with extraction occurring predominantly from one pool during the time course of the βCD. In general, we present the modified lattice model as an alternative option for geometric arrangements of the lipids in bilayers.

## Supplementary Materials

Supplementary File 1
