# Supplementary material for: Cholesterol Organization in Phosphatidylcholine Liposomes: A Surface Plasmon Resonance Study
Source: Materials (Basel). 2012 Nov 13;5(11):2306–25. doi: 10.3390/ma5112306 (PMC5448994; doi:10.3390/ma5112306)
Supplement: Supplementary File 1 [file materials-05-02306-s001.pdf]

Supplementary Information

Figure S1. The traditional model for arrays of cholesterol in phospholipids [1–3], in which red circles representing the acyl chains are placed in a hexagonal close packed array (**a**) and are then replaced with black circles representing the cholesterol (**b**). The cholesterol is shown here in a regular array that can be formed at a mole fraction $\chi_C = 0.5$. In this model, addition of cholesterol to a fixed amount of other lipids will increase the total area occupied by the lipids.

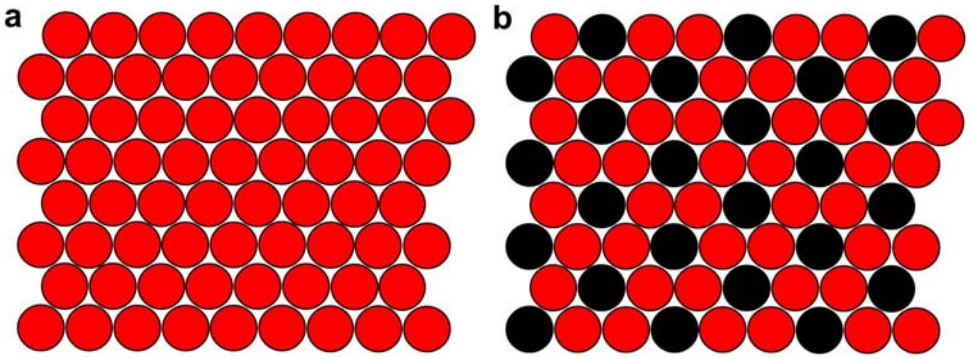

Figure S2. Representative raw data is shown for a cycle of liposome deposition ($\chi_C = 0.1$) and βCD addition on the modified surface of the SPR slide, during the course of one experiment (**a**). The addition times for the different solutions are indicated by arrows on the figure. A representative cycle is also shown in (**b**) for a series of depositions ($\chi_C = 0$–35), with βCD addition. At the end of each cycle, there is a detergent rinse followed by a buffer rinse which is intended to regenerate the surface of the acoustic device, so that a fresh layer of liposomes can be deposited. The features of the data that were analyzed were the net change in the SPR signal on addition of liposomes, the net drop in signal after addition of the βCD and the rate of decrease of the signal after addition of βCD.

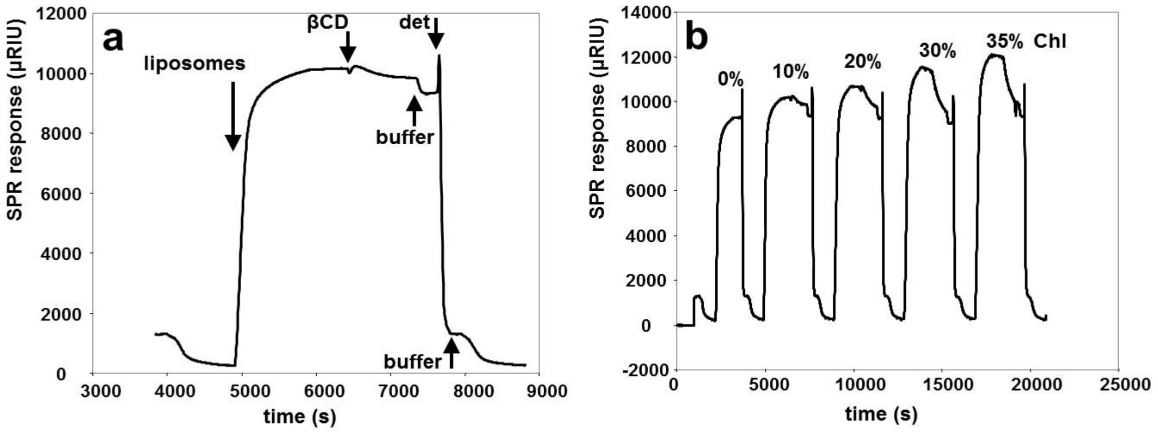

Figure S3. Regular arrays for χ_C = (**a**) 0.66; (**b**) 0.57; (**c**) 0.50; (**d**) 0.40; (**e**) 0.25; (**f**) 0.14. The arrays follow the model described in Figure 2.

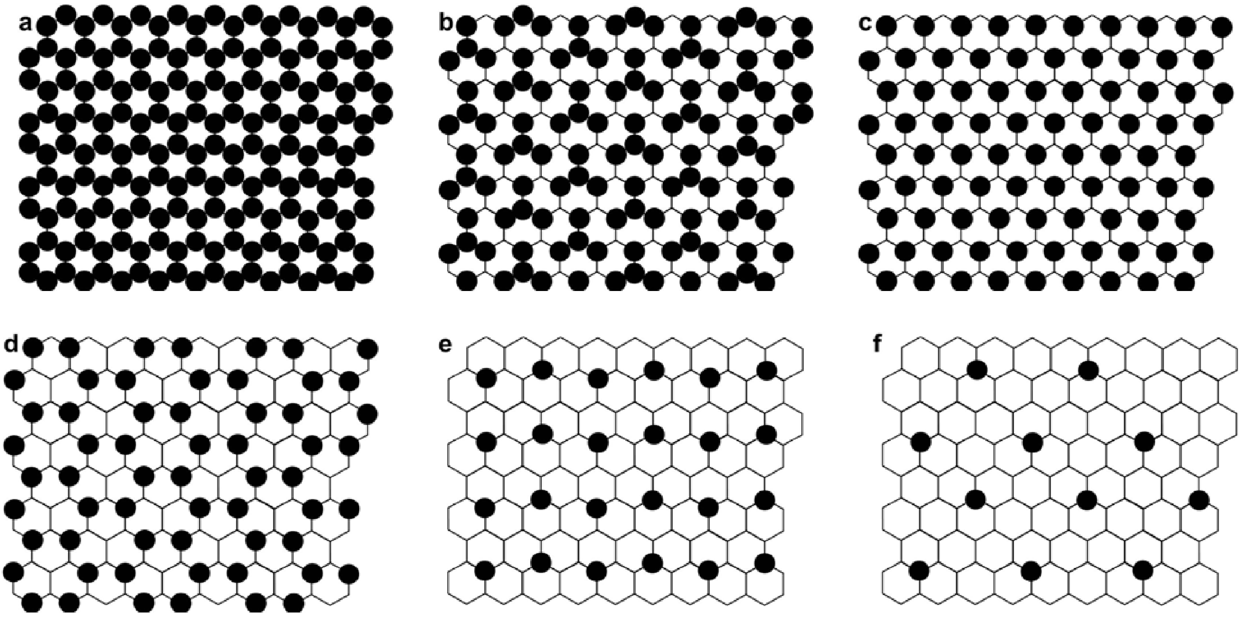

Figure S4. Kinetics of cholesterol removal from OPPC liposomes saturated with cholesterol, as for figure 7 but showing examples from individual experiments rather than averages from a series. The half-life times for the slow pool cholesterol are 312 s (**a**) and 292 s (**b**). The calculated mol% of cholesterol remaining in the liposomes is shown by the line in dark blue (see right-hand axis). The calculated χ_C remaining in the adsorbed liposomes is indicated on the figure for the filled coloured circles; colours correspond to those in Figure 5.

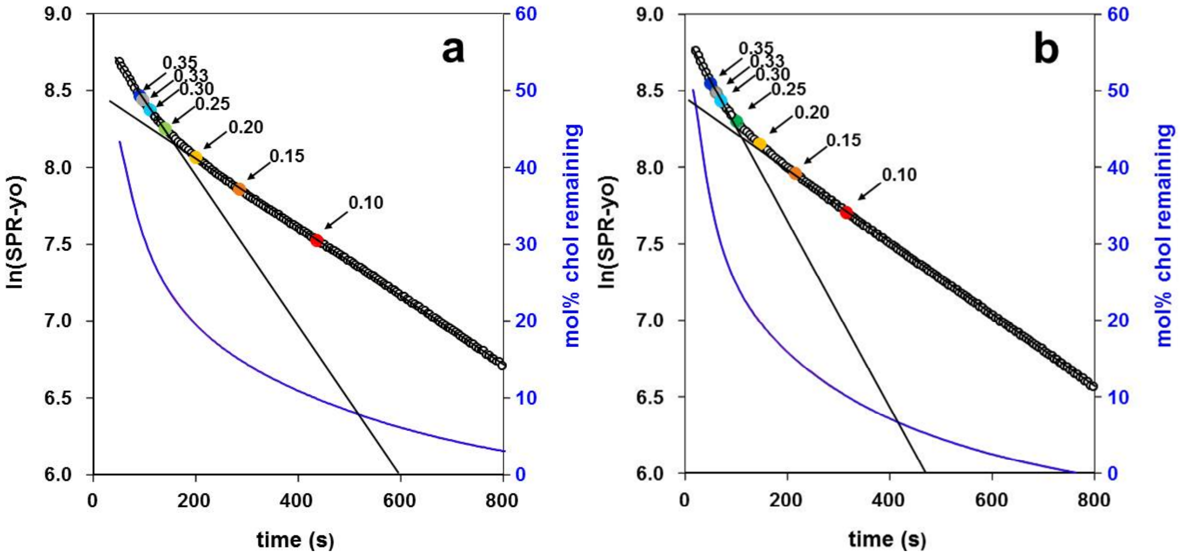

Figure S5. Cholesterol organization in phosphatidylcholine liposomes.

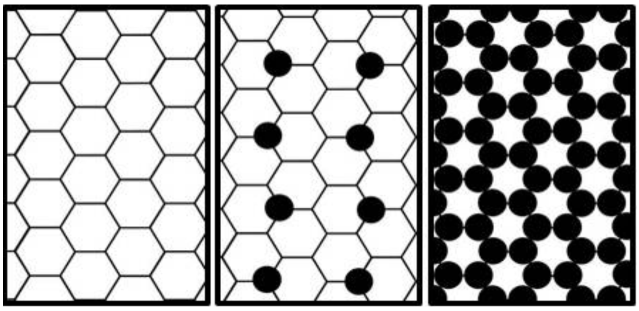

References

1. Ali, M.R.; Cheng, K.H.; Huang, J. Assess the nature of cholesterol-lipid interactions through the chemical potential of cholesterol in phosphatidylcholine bilayers. *Proc. Nat. Acad. Sci. USA* **2007**, *104*, 5372–5377.
2. Chong, P.L-.G. Evidence for regular distribution of sterols in liquid crystalline phosphatidylcholine bilayers. *Proc. Nat. Acad. Sci. USA* **1994**, *91*, 10069–10073.
3. Tang, D.; van der Meer, B.W.; Chen, S.Y. Evidence for a regular distribution of cholesterol in phospholipid bilayers from diphenylhexatriene fluorescence. *Biophys. J.* **1995**, *68*, 1944–1951.

© 2012 by the authors; licensee MDPI, Basel, Switzerland. This article is an open access article distributed under the terms and conditions of the Creative Commons Attribution license (http://creativecommons.org/licenses/by/3.0/).
